# Origin and Reversion of Omicron Core Mutations in the Evolution of SARS-CoV-2 Genomes

**DOI:** 10.3390/v15010030

**Published:** 2022-12-21

**Authors:** Xinwei Zhao, Luyao Qin, Xiao Ding, Yudi Zhang, Xuefeng Niu, Feng Gao, Taijiao Jiang, Ling Chen

**Affiliations:** 1 State Key Laboratory of Respiratory Disease, Guangdong Provincial Key Laboratory of Biocomputing, Guangzhou Institutes of Biomedicine and Health, Chinese Academy of Sciences, Guangzhou 510530, China; 2 Institute of Systems Medicine, Chinese Academy of Medical Sciences & Peking Union Medical College, Beijing 100005, China; 3 Suzhou Institute of Systems Medicine, Suzhou 215123, China; 4 State Key Laboratory of Respiratory Disease, Guangzhou Institute of Respiratory Health, the First Affiliated Hospital of Guangzhou Medical University, Guangzhou 510120, China; 5 Institute of Molecular and Medical Virology, Guangdong Provincial Key Laboratory of Virology, Institute of Medical Microbiology, School of Medicine, Jinan University, Guangzhou 510632, China; 6 Guangzhou Laboratory, Guangzhou 510005, China

**Keywords:** SARS-CoV-2, Omicron origin, core mutation, reverse

## Abstract

Genetic analyses showed nearly 30 amino acid mutations occurred in the spike protein of the Omicron variant of SARS-CoV-2. However, how these mutations occurred and changed during the generation and development of Omicron remains unclear. In this study, 6.7 million (all publicly available data from 2020/04/01 to 2022/04/01) SARS-CoV-2 genomes were analyzed to track the origin and evolution of Omicron variants and to reveal the genetic pathways of the generation of core mutations in Omicron. The haplotype network visualized the pre-Omicron, intact-Omicron, and post-Omicron variants and revealed their evolutionary direction. The correlation analysis showed the correlation feature of the core mutations in Omicron. Moreover, we found some core mutations, such as 142D, 417N, 440K, and 764K, reversed to ancestral residues (142G, 417K, 440N, and 764N) in the post-Omicron variant, suggesting the reverse mutations provided sources for the emergence of new variants. In summary, our analysis probed the origin and further evolution of Omicron sub-variants, which may add to our understanding of new variants and facilitate the control of the pandemic.

## 1. Introduction

The continuous evolution of SARS-CoV-2 resulted in new mutations and variants, causing a significant threat to global public health [1,2,3]. The World Health Organization (WHO) defined five variants of concern (VOCs) of SARS-CoV-2, and Omicron is currently the most infectious variant in the world [4]. Researchers identified five main sub-variants of Omicron, including BA.1, BA.2, BA.3, BA.4, and BA.5 [5] of which BA.1 was a typical Omicron and had more than 30 amino acid mutations in the spike protein [6]. Omicron showed stronger infectivity and immune evasion than other VOCs, which affected the host immune response and vaccine efficacy [7,8,9]. For example, G339D, K417N, G446S, E484A, N501Y, and Y505H mutations in the spike protein of Omicron could improve the evasion of antibody neutralization [10].

There are several inferences regarding the sudden appearance of Omicron and its sub-variants [11,12,13], such as (1) long-term evolution of SARS-CoV-2 in chronically infected/immunodeficient individuals; (2) low vaccination rates in Africa; (3) adaptive mutations in unknown animal hosts; and (4) new variants going unreported due to low levels of sequencing and detection ability in some countries. These undetermined factors make it difficult to trace back to the initial infected person or event. Researchers tried to study the origin and evolution of Omicron since its emergence, but it still needs to be clarified [13,14].

A large amount of available SARS-CoV-2 genomic data [15] (over 10 million genomes) makes it possible to explore the origin and evolution of Omicron. Comparative genomics and phylogenetic analysis approaches have been used extensively in previous SARS-CoV-2 tracing and evolutionary studies [16,17,18,19,20,21]. The genomic analysis showed 20 core mutations in the spike protein were shared in the sub-variants, BA.1–BA.5, of Omicron. This study detected associations between these core mutations by correlation analysis. Furthermore, the haplotype network was used to trace the emergence order of these core mutations and to infer the evolutionary pathway of Omicron, which was finally verified by epidemiological information and evolutionary tree analysis. Some sites of haplotype genomes experienced wild-type >> Omicron-mutation-type >> wild-type changes. Therefore, we call the latter Omicron-mutation-type to wild-type step the reverse mutation or reverse pathway.

## 2. Materials and Methods

### 2.1. Data Collection

The SARS-CoV-2 genomes and annotation data were downloaded from the GISAID and COVID-19 Viral Genome Analysis Pipeline databases [15,22]. Some mutations that are only in a specific sub-variant would not be representative of the whole Omicron variant population. Here, intersecting mutations of Omicron sub-variants are applied. A total of 20 mutations in the spike were found in all representative genomes of Omicron BA.1, BA.2, BA.3, and BA.4/5 compared to the wild type [Supplemental Table S1]. Representative genomes and site information were collected from the CoV-RDB and COVID-19 Viral Genome Analysis Pipeline databases [22,23]. These 20 characteristic mutations were also categorized by the outbreak database [24]. In this study, a total of 6,731,516 genomes sampled from 1st April 2020 to 1st April 2022 were analyzed, which did not have a gap in these 20 spike amino acid sites: 142, 339, 373, 375, 417, 440, 477, 478, 484, 498, 501, 505, 614, 655, 679, 681, 764, 796, 954, and 969.

### 2.2. Haplotype Network Construction

A total of 6,731,516 genomes were categorized based on the 20 core amino acid mutation sites of Omicron BA.1–BA.5. For each site, three types of amino acids were considered: (1) matching to the amino acid of wild type; (2) matching to the amino acid of Omicron; and (3) other type amino acid residue. There were theoretically 3^20^ possible haplotypes. Here, 93 haplotypes, including 6,692,471 (99.4%) genomes, were used to construct the network, where each haplotype had more than 500 sequences. The haplotype network was based on the information on these 20 sites, haplotype category information, and the number of haplotype members. The haplotype network was constructed by PopART v1.7 [25] and was visualized by Cytoscape v3.8.2 [26]. We defined the Omicron haplotypes with these 20 mutations as the intact-Omicron. Other Omicron haplotypes existing before and after the intact-Omicron category were defined as pre-Omicron and post-Omicron, respectively.

### 2.3. Time of Haplotype Genomes

We annotated the genome with the haplotype category and its sampling time. For genomes with the same haplotype category, the median sampling time is estimated as the time of the whole haplotype population. Each genome’s sampling time and haplotype were recorded and calculated by R v4.1.1 [27] to detect the overall time rank of haplotypes. The line plot of these data was drawn by the ggplot2 package [28] in R with the geom smooth function. Then, the mean sampling time for each haplotype population was calculated by R and shown in box plots.

### 2.4. Phylogenetic Tree Construction of Omicron Haplotypes

We annotated the genome with the haplotype category and submitted time information. The first submitted genome was selected as representative for genomes with the same haplotype annotation. There are 93 SARS-CoV-2 haplotypes of which 27 haplotypes are Omicron variants [Supplemental Table S2]. To focus on Omicron, we used these Omicron haplotypes and other VOCs. SARS-CoV-2 wild type (Wuhan/WIV04/EPI_ISL_402124), Alpha (B.1.1.7/EPI_ISL_1000001), Beta (B.1.351/EPI_ISL_1005538), Gamma (P.1/EPI_ISL_1000993), and Delta (AY.4/EPI_ISL_10004745) were used as outgroups. Then, these 32 sequences were aligned using FFT-NS-2 in MAFFT v7.487 [29]. The maximum likelihood tree was constructed by iqtree v2.1.3 [30] with 1000 bootstraps, where the best-fit nucleotide substitution model was chosen according to ModelFinder, and the tree was visualized by FigTree v1.4.4 [31]. Ancestral sites of Omicron haplotype trees were detected by MEGAX [32]. Then, the nucleic acid site information was manually converted to amino acid site mutations and reverses. Omicron core mutations and major reverses in the spike were labeled on branch nodes.

### 2.5. Correlation Analyses

In the correlation analysis, the amino acid matching to wild type was set to value 0; the amino acid matching to Omicron type was set to value 1; the others were set to value 0.5. Then, a matrix of 6,731,516 genomes × 20 sites was obtained. The Pearson correlation between every two sites was calculated by R [27]. The heatmap of Pearson correlation values was shown by the ggplot2 package [28] in R. With the same method, the Pearson correlation between every two haplotypes was calculated and plotted.

## 3. Results

### 3.1. The Core Mutations in the Spike of Omicron BA.1–BA.5

**Figure 1:**
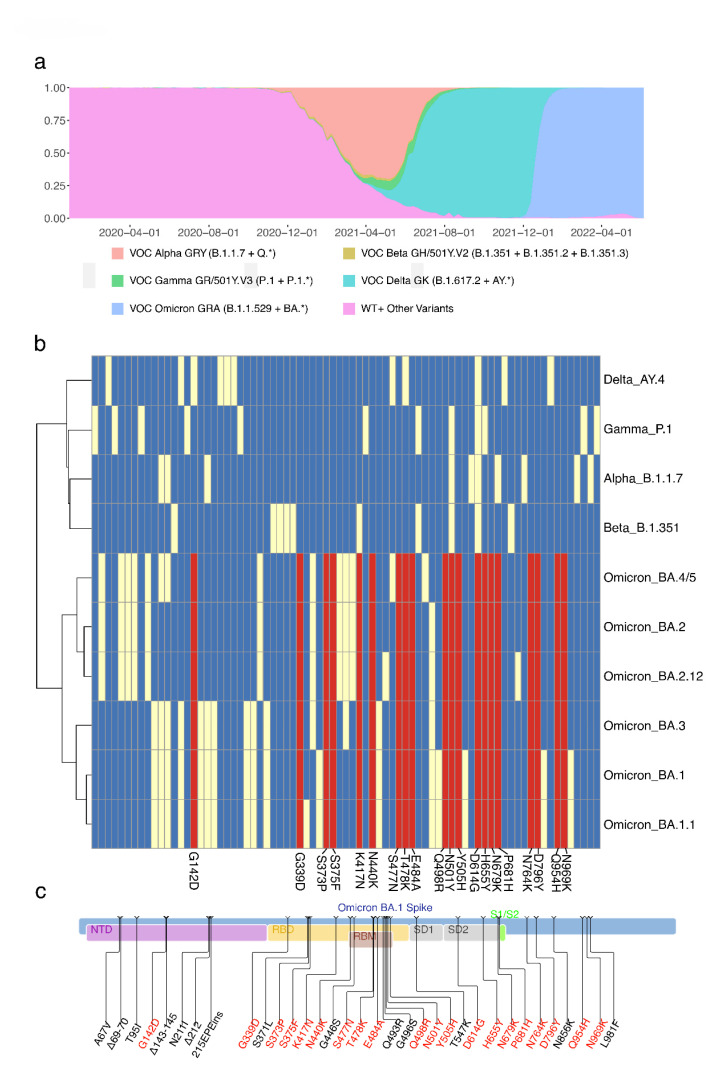
Twenty core mutations in the spike protein of Omicron variants. (**a**) The proportion of SARS-CoV-2 variants of concern (VOCs). (**b**) Mutations in the spike protein of VOCs. Red boxes are the spike amino acid mutations shared in Omicron sub-variants; yellow boxes are other spike amino acid mutations; blue boxes are wild types. (**c**) The positions of 20 core mutations in the spike of Omicron BA.1. Mutations with red color are the shared mutations in Omicron sub-variants.

There are four main waves in the spread of SARS-CoV-2: Wild type (WT), Alpha, Delta, and Omicron (Figure 1a). Based on representative genomes and mutations [15,22,23], genetic analysis showed over 70 amino acid sites had mutations in the spike protein of SARS-CoV-2, which were mainly distributed in the VOCs, including Alpha, Beta, Gamma, Delta and Omicron (Figure 1b,c). The sub-variants, BA.1 to BA.5, of Omicron shared 20 spike amino acid mutations: One mutation in the N-terminal domain (NTD) (G142D), eleven mutations in the receptor binding domain (RBD) (G339D, S373P, S375F, K417N, N440K, S477N, T478K, E484A, Q498R, N501Y, and Y505H), four mutations in SD (D614G, H655Y, N679K, and P681H), and four mutations in S2 (N764K, D796Y, Q954H, and N969K). In the outbreak database, only these 20 mutations are still characteristic mutations for the Omicron variant as of 24 October 2022 [24]. We focused on the characteristic mutations of the overall Omicron variant rather than its sub-variants. Therefore, we used only these 20 mutations shared by all sub-variants of BA.1, BA.2, BA.3, and BA.4/5. Some other mutations were necessary for specific sub-variants but were not representative of the Omicron variant. With the information on these 20 characteristic mutations of the Omicron variant, we could focus on the main pathway of Omicron origin and reversion. The presence of these mutations provides the basis for the emergence of Omicron.

### 3.2. The Evolutionary Pathway of SARS-CoV-2 Visualized by Haplotype Network

**Figure 2:**
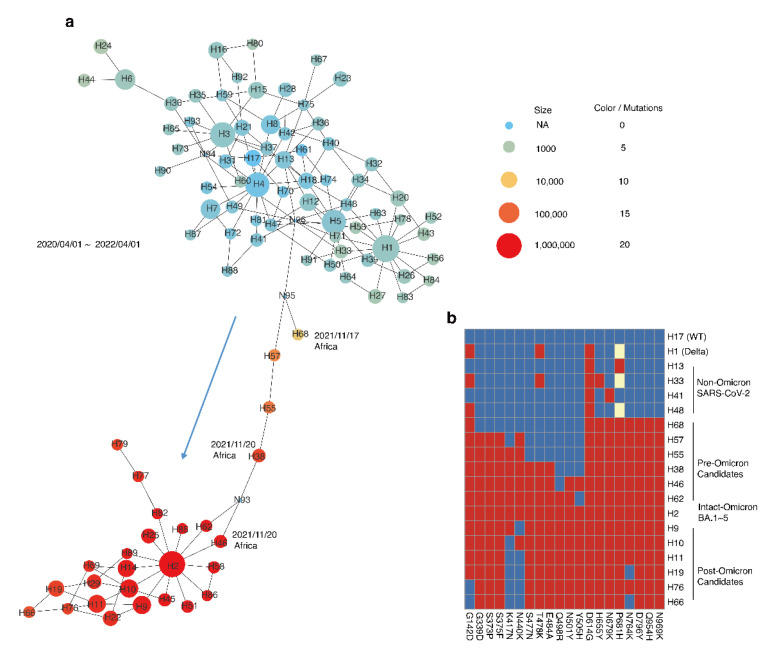
Haplotype networks of SARS-CoV-2 and the flow of Omicron core mutations. (**a**) Haplotype network of 20 core sites with genomes collected from 2020/04/01 to 2022/04/01. Node sizes represent the number of haplotype members; the color represents the number of omicron core mutations (blue–yellow–red: 0–10–20 core mutations); edges indicate the mutation steps. Haplotypes are named by their size rank from large to small: H1 to H93. N93 to N96 are undetected haplotypes. The arrow indicates the inferred order of haplotypes. (**b**) The profiles of 20 core mutations in the spike protein of Omicron. Red boxes are the core mutation types of Omicron; yellow boxes are the other amino acid mutation types; blue boxes are the wild types.

A total of 6.7 million SARS-CoV-2 genomes were categorized by the amino acid type of these sites. The haplotype network of the sequences collected from 1st April 2020 to 1st April 2022 is presented in Figure 2a, showing the interactions of 93 representative haplotypes, each with more than 500 sequences. The network also showed the number of core mutations, indicating the SARS-CoV-2 evolutionary trajectory in Figure 2a was from top to bottom. Combined with the epidemiology information, the haplotypes on the downside correspond to Omicron, where the haplotype H2 was intact-Omicron with 20 intact core amino acid mutations. The network showed the haplotypes, H68, H57, H55, H38, H46, and H62, were intermediates of H2. Although H68 is nearly a dead end in the haplotype network, it may provide features about Omicron in the early stage. In addition, epidemiological information indicated the ancestral close-related node, H68, was first detected on 17th November 2021 in Gauteng, South Africa. Then, H38 and H46 were found on 20th November 2021 in the same area.

As shown in Figure 2b, the amino acid mutation profiles of the main haplotypes revealed the accumulation process of 20 core mutations in the pre-Omicron candidates and the reversion of some core mutations in the post-Omicron candidates. Specifically, one or two core mutations occurred in non-Omicron SARS-CoV-2. Nearly half of the core mutations occurred and accumulated new core mutations in the pre-Omicron candidates until intact-Omicron BA.1–BA.5 appeared. Subsequently, some core mutations reversed to ancestral residues in the post-Omicron candidates, such as 142G, 417K, 440N, and 764N.

### 3.3. The Connection of Core Mutations in Omicron and Haplotypes

**Figure 3:**
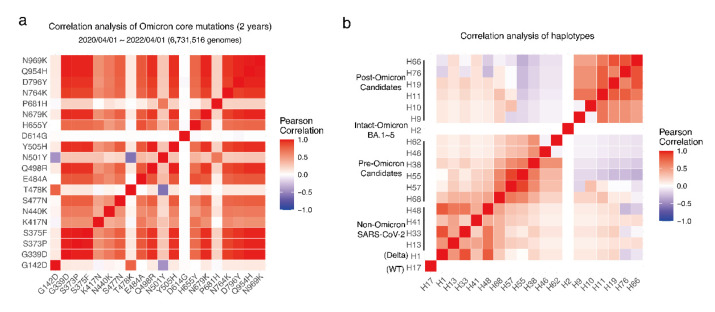
Correlation of Omicron core mutations and haplotypes. (**a**) Correlation analysis of Omicron core mutations based on the genomes collected from 2020/04/01 to 2022/04/01. (**b**) Correlation analysis of haplotypes. The color is scaled by the Pearson correlation of Omicron core mutation pairs (blue–white–red: −1–0–1).

The connections between mutations and between haplotypes were detected using correlation analysis (Figure 3a,b). Core mutations had tight correlations. Haplotypes in the non-Omicron were nearly correlated with each other, as well as the haplotypes in pre-Omicron and post-Omicron.

### 3.4. The Evolutionary Direction of Haplotypes in Omicron

**Figure 4:**
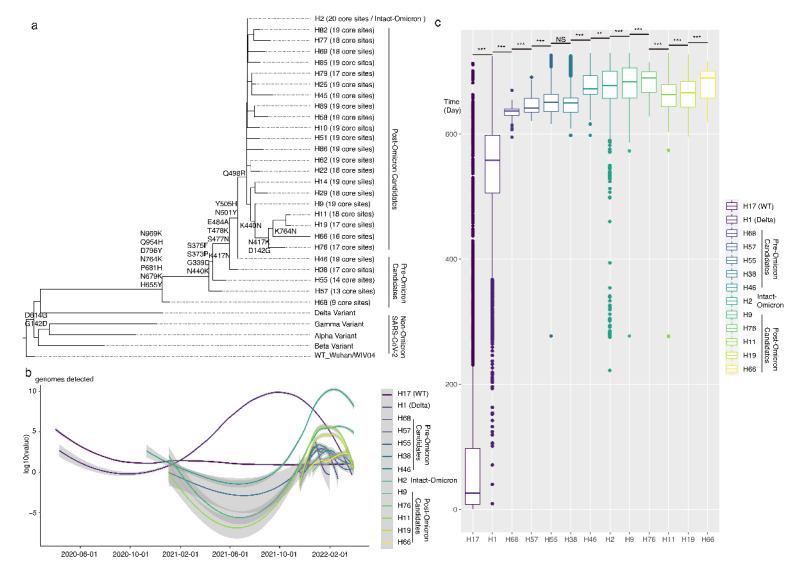
The evolutionary history and timeline of Omicron haplotypes. (**a**) Phylogenetic tree of Omicron haplotypes. Whole genomes of representative sequences of Omicron haplotypes were applied for tree construction. Tip labels are haplotype names of Omicron. The wild-type, Alpha, Beta, Gamma, and Delta variants are regarded as outgroups. The core mutations and major reverses in the spike of Omicron are labeled on branch nodes. (**b**) The detected number of SARS-CoV-2 genomes in each haplotype. The x-axis is the collected date; the y-axis is the log transferred genome number. Line shapes are loess smoothed. (**c**) Box plots show the collection time of all detected genomes in each haplotype. The x-axis is haplotypes; the y-axis shows the days between the collection time and 2020/04/01. The lines in the boxes are median values. *T* tests between haplotypes are marked with ***(*p* < 0.001), **(*p* < 0.01), and NS (not significant).

A phylogenetic tree, including the representative sequences of each haplotype, was constructed (Figure 4a). The branch length showed the pre-Omicron haplotypes (H68, H57, H55, H38, H46) were closer to the root (WT, WUHAN, EPI_ISL_402124) than intact Omicron haplotype H2. Moreover, many haplotypes in post-Omicron, such as H66 H11, H19, H76, and H9, showed longer evolutionary distances from the root. This result matched the previous haplotype network result. Based on the network analysis and phylogenetic tree analysis, we inferred the main direction of haplotypes is non-Omicron haplotypes >> H68 or H57 >> H55 >> H38 >> H46 >> H2 (with 20 intact Omicron core mutations) >> H9 >> H76 >> H11 or H19 >> H66.

Moreover, we checked the time of haplotype lineages to provide direct evidence and verify the inference above. In Fig 4b and 4c, H17, H1, H68, H57, H55, H38, H46, H2, H9, and H76 haplotypes appeared one after another as time went on, which was consistent with the inference, except H11, H19, and H66. Finally, we determined the pathway of formation and reversion of Omicron core mutations based on the haplotype network, phylogenetic tree, and epidemiological information.

### 3.5. Reverse Mutations in Omicron

**Figure 5:**
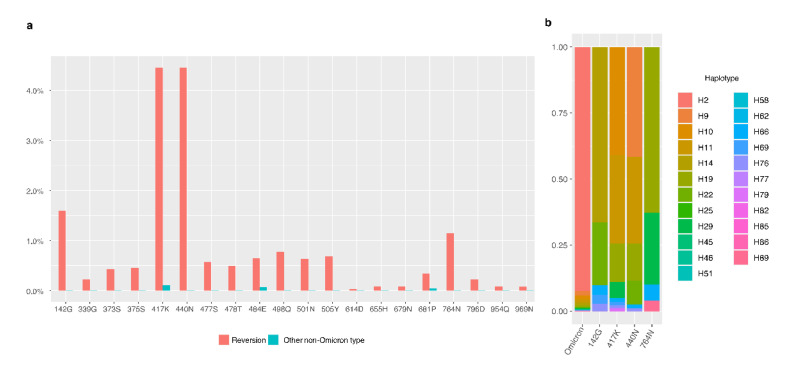
The proportion of reversions at the core sites of Omicron. (**a**) Revision of each core site of Omicron. Orange bars are the proportions of reversion in each core site. Blue bars are the proportions of the other non-Omicron-type mutations in each core site. (**b**), Intact-Omicron and post-Omicron haplotypes and their proportion on 142G, 417K, 440N, and 764N. Colors indicate different haplotypes.

As shown in Figure 2b, some core mutations reversed the ancestral residues in post-Omicron. Thus, we calculated the reversion frequencies of 20 core mutations (Figure 5) and found 417N and 440K had the highest reversion proportions, both at 4.45%, to reverse to ancestral residues, 417K and 440N, followed by 142D and 764K with 1.59% and 1.15% reverse to ancestral residues, 142G and 764N. These four sites were found in the post-Omicrons, such as the H66 haplotype, suggesting the reverse mutations provided material for the further emergence of new variants.

## 4. Discussion

In this study, we analyzed the evolution of the Omicron variants and the formation of 20 core amino acid mutations in the spike protein. There are approximately 43 days from H68 (pre-Omicron, nine core mutations) to H2 (intact-Omicron, twenty core mutations), which indicates the rapid formation of intact-Omicron is stepwise but abrupt. Since the main intermediate Omicron haplotypes and core mutations are traceable in the human population, the formation of intact-Omicron is highly possible in humans rather than animals. Indeed, no spillback to humans was detected in a study of SARS-CoV-2-infected free-ranging deer [33]. It has been reported there was no higher rate of evolution of SARS-CoV-2 lineages circulating in mink and deer than in humans [34].

Previous studies provided some speculative reasons for Omicron’s sudden appearance [9,10,11]. This research found only approximately 1/124 cases were sequenced per day from 2020/04/01 to 2022/04/01. The low-detection rate may lead to a deviation in estimating the time in the appearance of variants. However, posterior global data could eliminate these systematic errors and provide relatively accurate timing of occurring haplotypes or variants. To better predict the genome emergence time in a natural state, the collection date was mainly considered in this article. Because the sample size was large and the sample collection times were filled in manually, a few data inevitably had sequencing or filling errors. Here, the threshold value of 500 members for the haplotype population in this paper was used to reduce the possible bias of the entire population. Only 20 sites were considered for each genome in the haplotype network. A reasonable number of members of each node is needed. If more sites are considered, the fewer members of nodes will shrink their reliability and even decrease the resolution of these 20 sites when many nodes are below the threshold. In the outbreak.info database [24], these 20 sites still showed a high prevalence (K417N, N440K sites over 80%, other 18 sites over 90%) in the Omicron variant as of 24 October 2022, which indicates their persistence in the current data.

Studies showed these core mutations were associated with increased fitness of SARS-CoV-2, and the S373P and S375F substitutions not only changed the side chain but also induced a change in the conformation of the main chain, which can disrupt the hydrogen bonding of the antibody to the hairpin ring [35,36,37]. In the post-Omicron candidates, the ancestral residues, 142G, 417K, 440N, and 764N, had high ratios. The 417K reversion leads to evasion of Omicron antibodies [38], indicating the possible privilege of reverse mutations for Omicron survival.

It is possible recombination caused revisions on sites 142, 417, 440, and 764 from Omicron-type to wild-type. However, the highly conserved spike sequences could hardly provide reliable resolution of recombination events for specific sites or small regions. Nevertheless, recombination or mutation sources will lead to the same revision characteristic consequence for Omicron spikes. More than half of the BA.2.75.9, BL.4, and BM.4 genomes had revisions at site 142, and BA.2.75.9 also had a revision at site 417 [24], indicating reversion also applies to recent lineages.

Studies showed the genetic distance between the vaccine and the virus correlates with vaccine efficacy [39]. The haplotype sequences identified in this study have intermediate genetic distances between the wild-type and the Omicron variant with intact core mutations, providing additional antigenic candidates for the design of broad-spectrum efficient vaccines. We hope this study can provide a holistic and dynamic perspective on the evolution of SARS-CoV-2 and the formation and development of Omicron and then provide a basis for vaccine design.

## Data Availability

The SARS-CoV-2 genome sequences, annotation, and mutation data used in this paper are publicly available from the GISAID database (https://www.gisaid.org/: accessed on 9 June 2022), COVID-19 Viral Genome Analysis Pipeline databases (https://cov.lanl.gov/: accessed on 11 July 2022), and CoV-RDB database (https://covdb.stanford.edu/: accessed on 11 July 2022).

## Supplementary Materials

The following supporting information can be downloaded at https://www.mdpi.com/article/10.3390/v15010030/s1: Table S1: Mutations of VOC and Omicron spikes; Table S2: Genome information of representative haplotypes.
